# Clinical and psychopathological features of parents of patients with anorexia nervosa.

**DOI:** 10.1192/j.eurpsy.2024.643

**Published:** 2024-08-27

**Authors:** O. Khaustova, L. Sak

**Affiliations:** ^1^Bogomolets National Medical University, Kyiv, Ukraine

## Abstract

**Introduction:**

Anorexia nervosa (AN) is a widespread chronic mental disorder with severe negative medical and social consequences. Treating patients with AN is a complex and time-consuming process, as persistent forms are often encountered. The studies’ results indicate a possible influence of psychoemotional state and/or existing psychopathological manifestations in parents on AN development.

**Objectives:**

To investigate the clinical and psychopathological characteristics of parents of patients with AN based on the study of emotional regulation, alexithymia, depression, and anxiety.

**Methods:**

The study population (N=110) consisted of fathers (N=47 (42.7%)) and mothers (N=63 (57.3%) of patients with AN. The mean age was M=44.90 (SD=5.9; SE=0.567). All participants completed the emotional regulation scale (DERS), Toronto Alexithymia Scale (TAS-26), Hospital Anxiety and Depression Scale (HADS).

**Results:**

According to DERS: “rejection” - 21.86 (SD=5.675; SE=0.541); “goals” - 19.13 (SD=2.028; SE=0.193); “impulse” - 24.17 (SD=4.908; SE=0.468); “awareness” - 21.93 (SD=1.999; SE=0.191); “strategies” - 30.75 (SD=2.173; SE=0.207); “clarity” - 18.58 (SD=1.486; SE=0.142). The sum was 136.42 (SD=8.119; SE=0.774). The TAS results of the study group were 80.45 (SD=13.699), which characterizes the average personality type as alexithymic. According to HADS, the average values were distributed: the anxiety scale M=7.96 (SD=1.347) the depression scale M=7.95 (SD=1.442). These indicators can be considered as the extreme limit of the norm or subclinically expressed anxiety and depression. The next step was to find statistically significant relationships between the DERS methodology and the HADS and TAS for the study group. According to Spearman’s correlation coefficient, there is a direct stable relationship between the variables “anxiety” and “impulse” (r=0.257), awareness (r=0.255), and the total score of emotional regulation according to “DERS” (r=0.246); A direct correlation was found between the indicators “depression” and “rejection” (r=0.151), “goals” (r=0.233), “awareness” (r=0.138); Alexithymia, in turn, has a direct correlation with the “goals” scale and an inverse correlation with the “strategies” scale (r=-0.141)

**Conclusions:**

Parents of patients with AN have various manifestations of psychoemotional disturbances, namely subclinical levels of depression and anxiety, high levels of alexithymia, and emotional regulation problems. The correlation analysis showed that the anxiety score for parents of patients with AN is higher if difficulties with impulse control, emotional awareness, and general emotional regulation are problematic. Depressive tendencies are also associated with the subjects’ rejection of emotional reactions and problems with goal-directed behavior. The inverse correlation indicates that the higher the index of alexithymia, the less limited access to emotion regulation strategies, and vice versa.

**Disclosure of Interest:**

None Declared

